# Genome-Wide Identification of the Soybean UMAMIT Family and Functional Analysis of *GmUMAMIT118* in Improving Seed Protein Content

**DOI:** 10.3390/ijms27167079

**Published:** 2026-08-07

**Authors:** Yongjiang Bi, Yaohui Chen, Meirong Lang, Li Duan, Pei Song, Yudong Yang, Xiangxiang Ye, Yan Liu, Bangjun Wang

**Affiliations:** Chongqing Key Laboratory of Plant Resource Conservation and Germplasm Innovation, Key Laboratory of Eco-Environments in Three Gorges Reservoir Region (Ministry of Education), College of Life Sciences, Southwest University, Chongqing 400715, China

**Keywords:** amino acid transporter, UMAMIT, soybean, gene family, seed protein content

## Abstract

Seed protein content, oil content, and yield are key agronomic traits that determine the economic value of soybean. For decades, soybean has served as a leading source of plant protein for human and animal nutrition due to its high protein concentration. Manipulating amino acid transporters to regulate the direction of nitrogen allocation represents a promising strategy for improving seed protein content. Multiple studies have employed this strategy by targeting amino acid importers. Recently, the Usually Multiple Amino acids Move In and Out Transporter (UMAMIT) family has been characterized as amino acid exporters; nevertheless, their role in regulating the seed protein content of soybean has not yet been investigated. In this study, we identified 120 soybean *UMAMIT* genes via a genome-wide search and designated them according to chromosomal location. Phylogenetic analysis grouped these genes into 10 clades (A–J). Whole-genome duplication (WGD)/segmental duplication served as the main driver of the GmUMAMIT family expansion, followed by tandem duplication. By integrating transcriptome data with QTL/GWAS loci, we identified twelve candidate genes associated with seed protein content and verified their expression patterns during seed development via qPCR. One candidate gene, *GmUMAMIT118*, was selected and overexpressed in *Arabidopsis thaliana*, resulting in transgenic lines with significantly higher seed protein content and yield. Collectively, these results provided a comprehensive overview of the soybean UMAMIT family and offered a preliminary exploration of its role in improving seed protein content.

## 1. Introduction

Nitrogen (N) is a fundamental nutrient for plant growth and reproduction [1]. In plants, amino acids serve as the predominant form of organic N for long-distance transport and source-to-sink partitioning. These amino acids, synthesized primarily in roots and mature leaves or released from senescent leaves, are carried to the sinks. The major N sinks are developing roots and leaves during vegetative growth, whereas during the reproductive stage, N is dominantly remobilized to flowers, fruits, and seeds [2]. This interorgan and intercellular flux of amino acids is critical for plant growth and development, and is primarily mediated by amino acid transporters.

The known plant amino acid transporters are classified into three families: the Amino Acid/Auxin Permease (AAAP) family, the Amino Acid-Polyamine-Organocation (APC) family, and the Usually Multiple Acids Move In and Out Transporter (UMAMIT) family [3]. The AAAP family includes amino acid permeases (AAPs), lysine/histidine transporters (LHTs), γ-aminobutyric acid transporters (GATs), proline transporters (ProTs), aromatic and neutral amino acid transporters (ANTs), and indole-3-acetic acid transporters (AUXs). The APC family comprises cationic amino acid transporters (CATs), amino acid/choline transporters (ACTs), and polyamine H+-symporters (PHSs) [4,5].

The AAAP and APC families are collectively known as amino acid transporters (AATs), and several members have been extensively studied. The first plant amino acid transporter identified, Arabidopsis AtAAP1, mediates amino acid uptake in embryos [6,7,8]; AtLHT1 is essential for the uptake of N in roots and the supply of leaf mesophyll with xylem-derived amino acids [9,10]; AtCAT2, located at the tonoplast, is involved in regulating leaf amino acid concentrations [11]. OsLHT1 plays a crucial role in N allocation in rice, and was recently shown to enhance organic nitrogen use efficiency through mediating amino acid absorption and interaction with the rhizosphere microbiota [12,13]. In legumes, overexpression of additional copies of *PsAAP1* in pea increased phloem loading and embryo loading of amino acids and the uptake of N in roots, resulting in significantly higher seed yield and greater seed protein productivity, as well as improved nitrogen use efficiency (NUE) [14,15]. Similarly, MtCATs are required for maintaining efficient symbiotic nitrogen fixation in *Medicago truncatula* [16]. In soybean, overexpression of *GmAAP6a*—which is primarily expressed in phloem and xylem parenchyma cells—enhances low-nitrogen tolerance and source-to-sink transport, thereby improving seed quality [17]. Additionally, overexpression of a GmAAP6-like transporter in Arabidopsis contributes to seed quality and yield [18].

The UMAMIT family, also known as the P-DME (plant Drug/Metabolite Exporter)/MtN21(Nodulin 21 of Medicago truncatula)/EamA-like family, is a class of transporters that can transport multiple acids in plants. The P-DME family was initially characterized as a plant-specific subset of the DME (Drug/Metabolite Exporter) family during the design of the Drug/Metabolite Transporter (DMT) superfamily [19]. DME family members share the conserved EamA domain, and some functionally characterized proteins can export amino acids [20,21,22]. The founding member, *MtN21* from *Medicago truncatula*, was first described as a gene induced during root nodulation [23]. A homolog of *MtN21* in loblolly pine, *5NG4*, was later found to be an auxin-inducible gene [24]. AtWAT1 (Walls are thin 1/AtUMAMIT5) is located at the tonoplast and is required for proper secondary cell wall formation in stems, and functions as a vacuolar auxin export facilitator [25,26]. Its tomato homolog SIWAT1 shares a similar function [27]. The first UMAMIT protein with clearly defined amino acid export activity was AtSIAR1 (Siliques Are Red 1/AtUMAMIT18), which plays an important role in organic nitrogen transport during silique development in Arabidopsis [28]. Since then, AtSIAR1 and its homologs have been designated as UMAMITs in the Arabidopsis TAIR database. Subsequent studies identified that several Arabidopsis UMAMITs, including AtUMAMIT11, 14, 18, 24, 25, 28, and 29, were shown to be facilitators of amino acid export in seeds [28,29,30]; AtUMAMIT14 is engaged in amino acid phloem unloading in roots [31]; AtUMAMIT14 and AtUMAMIT18 participate in radial amino acid transport within roots [31]. Recently, AtUMAMIT44 was shown to control glutamate export from chloroplasts and to be vital for nitrogen partitioning from leaves to sinks [32]. Loss-of-function *atumamit30* mutants exhibit compromised amino acid root exudation, but accumulate amino acids in shoots, suggesting a defect in phloem loading [33]. UMAMITs also influence plant–pathogen interactions; the *atwat1* mutant conferred broad-spectrum resistance against vascular pathogens [34], and the *atrtp1* (*Resistance to Phytophthora parasitica 1/AtUMAMIT36*) mutant displayed increased resistance to biotrophic pathogens [35]. Overexpressing *AtUMAMIT14* increases salicylic acid levels and enhances pathogen resistance [36]. AtUMAMIT20 accumulates in leaf vasculature close to the site of *Botrytis cinerea* infection, and *atumamit20* mutants exhibit decreased susceptibility [37]. Research has also revealed that a brassicaceous-specific clade of UMAMITs is able to export specialized metabolites: AtUMAMIT29, 30, and 31 function as glucosinolate exporters, and UMAMIT exporters and GTR importers form an essential transporter cascade for translocating glucosinolates into seeds [38,39,40]. Interestingly, recent studies also linked UMAMITs to their role as amino acid exporters in symbiotic nitrogen fixation; the *mtumamit* mutants do not show altered infection capacity, but show reduced nitrogen-fixing symbiosis [41,42]. Although named “Usually Multiple Acids Move In and Out Transporter”, most functionally characterized UMAMITs act as uniporters that export amino acids, whereas AAAP and APC family members typically function as importers [43]. This export function positions UMAMITs with great potential as the key to the ‘missing link in nitrogen cycling’ [4]. Single-cell RNA sequencing of *Arabidopsis thaliana* and *Zea mays* leaf phloem cells revealed that SWEET (Sugars Will Eventually Be Exported Transporter) sugar transporters and UMAMIT amino acid transporters share high co-expression levels. Some UMAMITs are specifically expressed in phloem parenchyma cells, indicating a key role in phloem loading [44,45,46].

Soybean (*Glycine max*) is a leading dual-function crop that provides highly valuable protein and oil, playing a crucial role in global food security [47]. Soybean seed protein is widely utilized in animal feed and food supplements, and is considered a sustainable, low-cost, and biodegradable alternative to petroleum-derived polymers [48]. Manipulating amino acid transporters to regulate source–sink partitioning or NUE has been proven to be an effective strategy for increasing seed protein yield [49,50]. Although UMAMIT family members have been identified in Arabidopsis and wheat and their evolutionary history has been examined by recent phylogenetic analyses [43,51,52], soybean UMAMIT family members remain uncharacterized. Here, we identified 120 putative members in soybean and proposed 12 candidate genes that may increase seed protein content. Furthermore, overexpressing *GmUMAMIT118* in Arabidopsis was shown to increase seed protein content and weight. These results provide a framework for further research on the soybean UMAMIT family and offer insight into soybean breeding.

## 2. Results

### 2.1. Identification of Putative UMAMIT Genes in Soybean

Two approaches were used to search the soybean genome: BLASTP searches using known *Arabidopsis thaliana* UMAMIT protein sequences as queries and HMM searches with the HMM profile of the EamA domain (PF00892). A total of 120 *UMAMIT* genes were identified and named *GmUMAMIT1–120* according to their chromosomal locations. Detailed information on GmUMAMIT members, including locus name, chromosomal position, protein length (amino acids), molecular weight, isoelectric point, and subcellular localization, is presented in Table S1. In summary, most GmUMAMIT proteins were basic proteins (isoelectric point, 8.5–10), with lengths of 300–400 amino acids and molecular weights of 35–45 kDa. The predicted subcellular localizations of GmUMAMITs were mainly in the plasma membrane, followed by the chloroplast.

### 2.2. Phylogenetic Analysis

**Figure 1:**
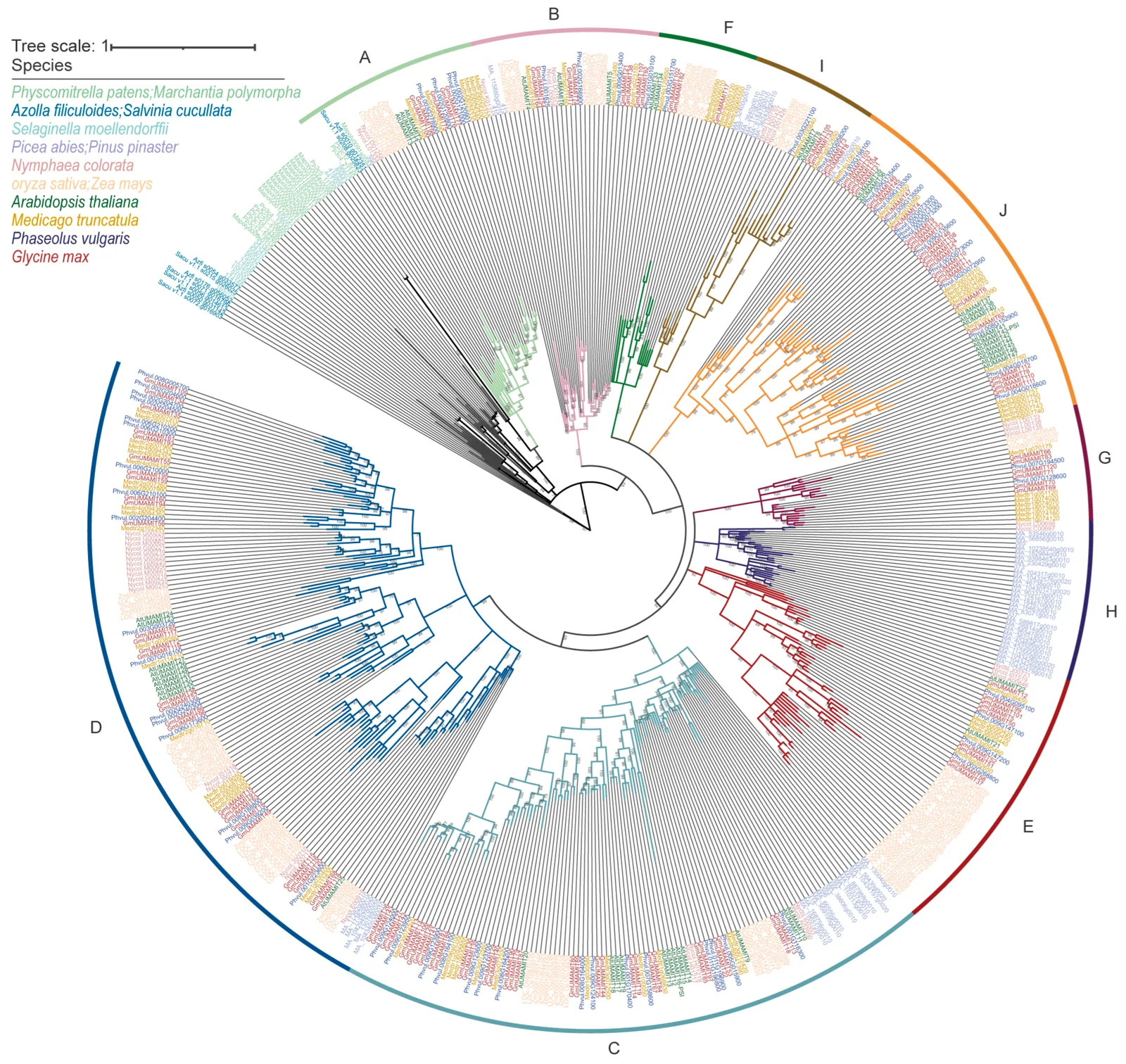
The phylogenetic tree was constructed using 549 UMAMIT proteins from soybean and 13 other representative species. According to Zhao et al. [43], all UMAMIT members were grouped into ten clades (A–J). The maximum likelihood tree was constructed using IQ-Tree, with the best model of JTT+F+R7, and UltraFast bootstrap was 5000 times. The accession number of the UMAMIT members of *Marchantia polymorpha*, *Physcomitrella patens*, *Selaginella moellendorffii*, *Azolla filiculoides*, *Salvinia cucullata*, *Nymphaea colorata*, *Pinus pinaster*, *Picea abies*, *Arabidopsis thaliana*, *Zea mays*, *Oryza sativa*, *Medicago truncatula*, *Phaseolus vulgaris* and *Glycine max* is listed in Table S2.

To investigate the phylogenetic relationships of the UMAMIT proteins in plants, we identified UMAMIT proteins from *Marchantia polymorpha* (13), *Azolla filiculoides* (4), *Salvinia cucullata* (6), *Nymphaea colorata* (37), *Oryza sativa* (54), *Zea mays* (46), *Medicago truncatula* (78), and *Phaseolus vulgaris* (62). Together with published UMAMIT proteins from *Physcomitrella patens*, *Selaginella moellendorffii*, *Arabidopsis thaliana* and the two conifers, *Pinus pinaster* and *Picea abies* (8, 13, 46, 16, and 4, respectively) [43], a total of 549 proteins from 14 representative plant species were used to construct a phylogenetic tree (Figure 1 and Table S2). Maximum likelihood analysis grouped the genes into 10 clades (A–J). Clade A contained members from almost all species, while clades C and D contained the largest number of members. All proteins from *M. polymorpha*, *P. patens*, *S. moellendorffii*, and two fern species, *A. filiculoides* and *S. cucullata*, were clustered in clade A and the outgroup. Clade H was mainly composed of conifer sequences, with only two sequences from *N. colorata*. Members of *N. colorata* were enriched in clade D. Gene duplication patterns in monocots and eudicots appeared to differ; *O. sativa* and *Z. mays* UMAMIT members were enriched in clades D and E, whereas no members were present in clade J. In contrast, members of *A. thaliana*, *Glycine max*, *M. truncatula*, and *P. vulgaris* were abundant in clades C, D, and J. Soybean contained 32, 29, and 24 members in clades C, D, and J, respectively, accounting for 70.83 percent of the total. Notably, soybean possessed 120 UMAMIT members, which was considerably more than those in other species and nearly double that of *P. vulgaris*. The phylogenetic tree comprising soybean and *A. thaliana* UMAMIT members is shown in Figure S1.

### 2.3. Characterization of Conserved Domain, Motif, and Gene Structure

**Figure 2:**
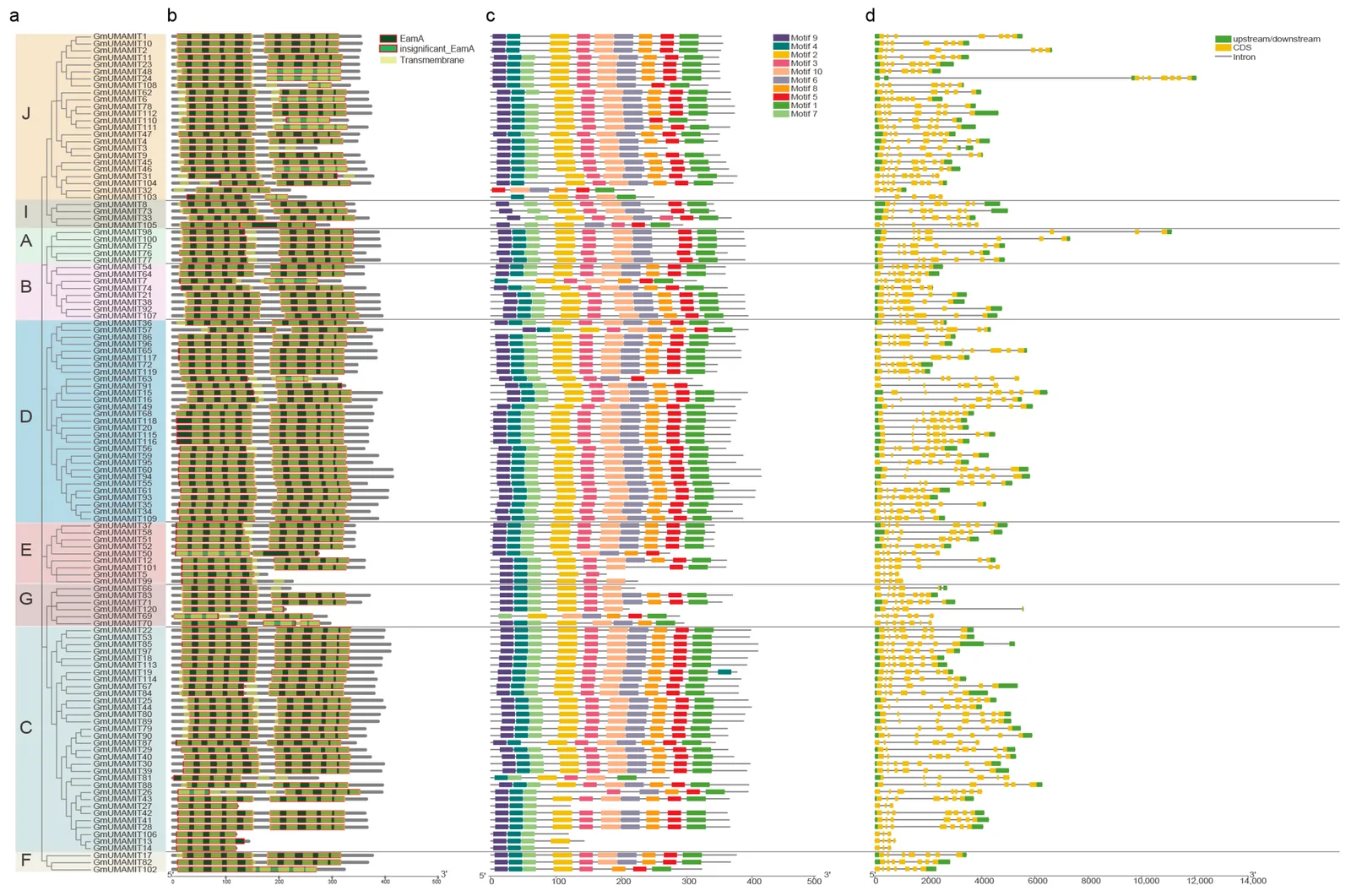
Analysis of conserved protein domains, motifs and gene structures of GmUMAMIT members along with the phylogenetic tree. (**a**) represents the phylogenetic tree (Clade A–G, I, J), (**b**) displays conserved protein domains, (**c**) displays conserved protein motifs and (**d**) shows the gene structures. Detailed information is given in Table S3.

To further elucidate the evolutionary relationships of soybean UMAMIT members, we investigated the conserved protein domains, motifs, and gene structures in conjunction with phylogenetic trees (Figure 2 and Table S3). Only EamA domains were identified in GmUMAMIT members, with the majority (91.7%, 110/120) consisting of dual EamA domains, of which at least one EamA domain was significant. All GmUMAMIT members in clade A contained dual significant EamA domains. Proteins that contained a single EamA domain were distributed in clades C, E, G, and J, mainly in C (50%, 5/10). CCTOP predicted that the number of transmembrane domains (TMDs) in GmUMAMIT members ranged from 4 to 10, with a predominance of 10. This pattern is consistent with the presence of dual EamA domains, as each EamA domain typically contains five TMDs. All TMDs were detected within the EamA domains at relatively constant intervals. TMDs were also identified in non-significant EamA domains or at the N- and C-termini of the domains, indicating that the topological structure was more conserved than amino acid residues. Consistently, the overall sequence similarity among GmUMAMIT proteins was relatively low (Figure S2 and Table S4). GmUMAMIT members typically contained 10 motifs, arranged in the order of 9, 4, 7, 2, 3, 10, 6, 8, 5, and 1. Motif 8 was absent from clade A, and clade I exhibited relatively low conservation. Although intron lengths varied considerably, the exon/intron number was typically 7/6. Some members of clades B and D showed fusion of exons 4 and 5. Notably, clade A exhibited a higher degree of conservation, with members showing consistency in motifs, domains, topology, and intron–exon organization. These observations suggest that the ancestral soybean *UMAMIT* gene contained seven exons and six introns, and that the encoded proteins were composed of dual EamA domains and contained ten TMDs.

### 2.4. Analysis of Chromosomal Distribution, Gene Duplication Events, and Ka/Ks Ratio

**Figure 3:**
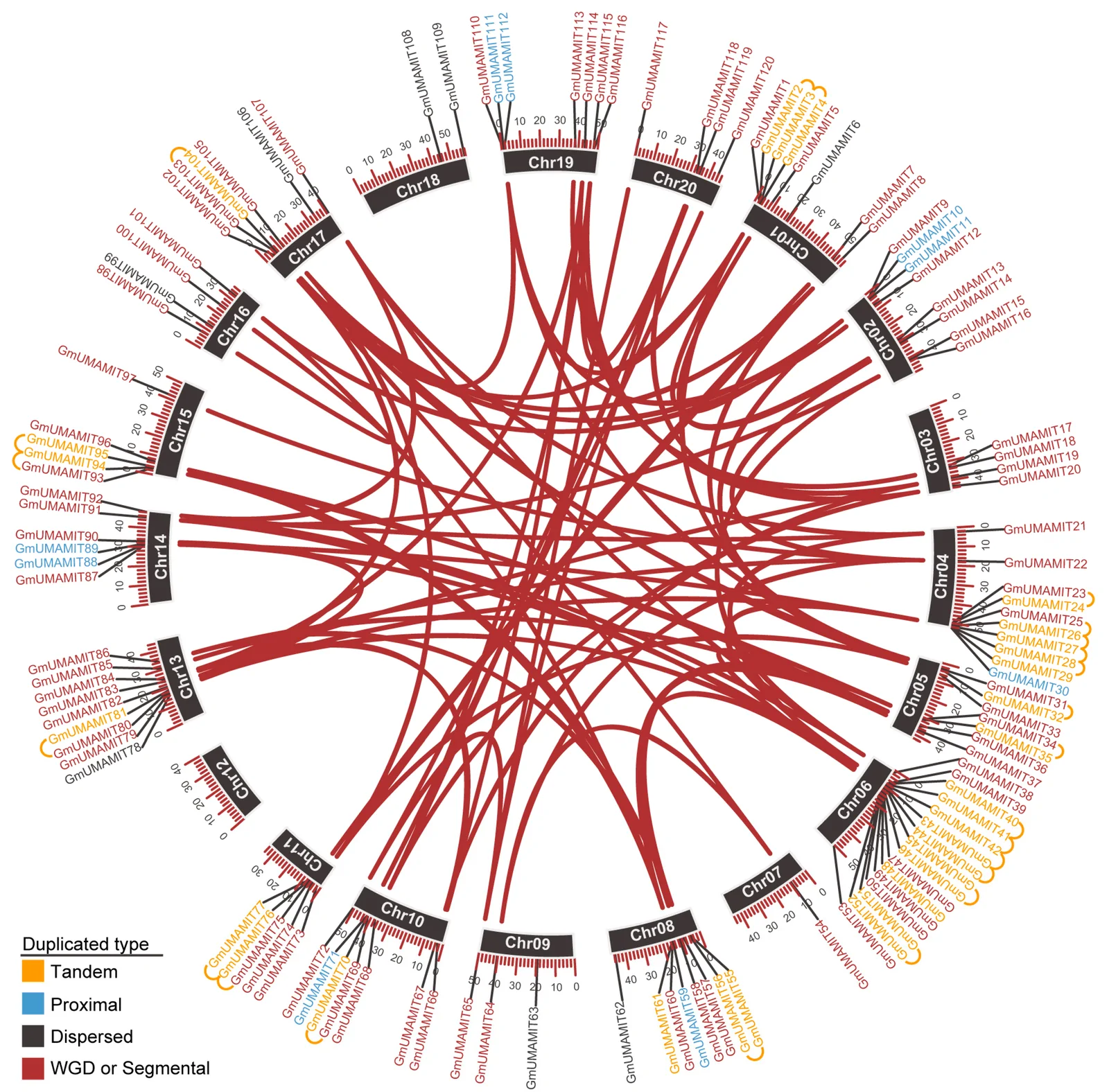
Gene duplication relationship among GmUMAMIT members. Red lines between chromosomes indicate WGD or segmental duplicate gene pairs, and orange lines between genes indicate tandem gene pairs. Detailed information is given in Table S5.

Gene duplication is widespread in plant genomes, and duplicated genes are the source for the origin and evolution of new genes, as well as the generation of functional novelty and specialization. Chromosomal locations of GmUMAMIT genes were mapped according to the GFF3 file, and collinearity was detected using MCScanX (Figure 3). The soybean genome contains 20 chromosomes, and 120 members were unevenly distributed across 19 chromosomes, with chromosome 12 lacking any members. The largest number of GmUMAMIT genes on a single chromosome was observed on chromosome 6. Four types of gene duplication were identified: whole-genome duplication (WGD) or segmental, tandem, dispersed, and proximal. More than half (73/120, 60.8%) of GmUMAMIT genes were generated by WGD or segmental duplication, followed by tandem duplication (30/120, 25.0%). These two mechanisms represented the major driving forces for GmUMAMIT gene family expansion (Table S5). Tandem duplicated genes were mainly present on chromosomes 4 and 6, whereas WGD/segmentally duplicated genes were present on all chromosomes except chromosome 12 (no members) and 18 (two dispersed members).

To investigate the WGD events of GmUMAMIT members, 78 WGD or segmental gene pairs and 22 tandem gene pairs were identified. We calculated the Ks, Ka, and Ka/Ks ratios of these gene pairs (Table S6). Due to the high sequence divergence (pS > 0.75), Ks values could not be obtained for four gene pairs. Seven gene pairs (Ks > 2) were excluded to avoid substitution saturation. The Ka/Ks ratios of all gene pairs were less than 1, indicating that GmUMAMIT genes have undergone strong purifying selection. Three rounds of WGD events have occurred during soybean genome evolution: the γWGD event approximately 130–240 million years ago (Mya), the legume WGD event approximately 58 Mya, and the Glycine genus WGD event approximately 13 Mya [53,54]. Divergence times were calculated for 78 pairs of collinear genes to analyze these WGD events (Tables S5 and S6). All three rounds of WGD events were detected, and most WGD events were attributed to the legume WGD and Glycine genus WGD, accounting for 52.24% and 32.84%, respectively. To better understand the reasons for the large number of members of the GmUMAMIT gene family, we detected genome-wide collinearity between *Glycine max* and *Arabidopsis thaliana*, *Oryza sativa*, *Medicago truncatula*, and *Phaseolus vulgaris* (Figure S3, Tables S5–S7). *Glycine max* UMAMIT members shared 76, 27, 107, and 122 collinear gene pairs with *A. thaliana*, *O. sativa*, *M. truncatula*, and *P. vulgaris*, respectively. Intraspecies collinearity in *M. truncatula* and *P. vulgaris* was further analyzed (Figure S3, Tables S5–S7). We identified 11 gene pairs within collinear blocks and 12 tandem gene pairs in *M. truncatula*, whereas 39 collinear gene pairs and 21 tandem gene pairs were identified in *P. vulgaris*; both totals were lower than those in *G. max*. A considerable proportion of gene pairs in *M. truncatula* and *P. vulgaris* were excluded because their Ks values were greater than 2. The Ka/Ks ratios of the remaining gene pairs were less than 1, and only γWGD and legume WGD events were detected in these species.

### 2.5. Analysis of Tissue-Specific Expression Patterns

**Figure 4:**
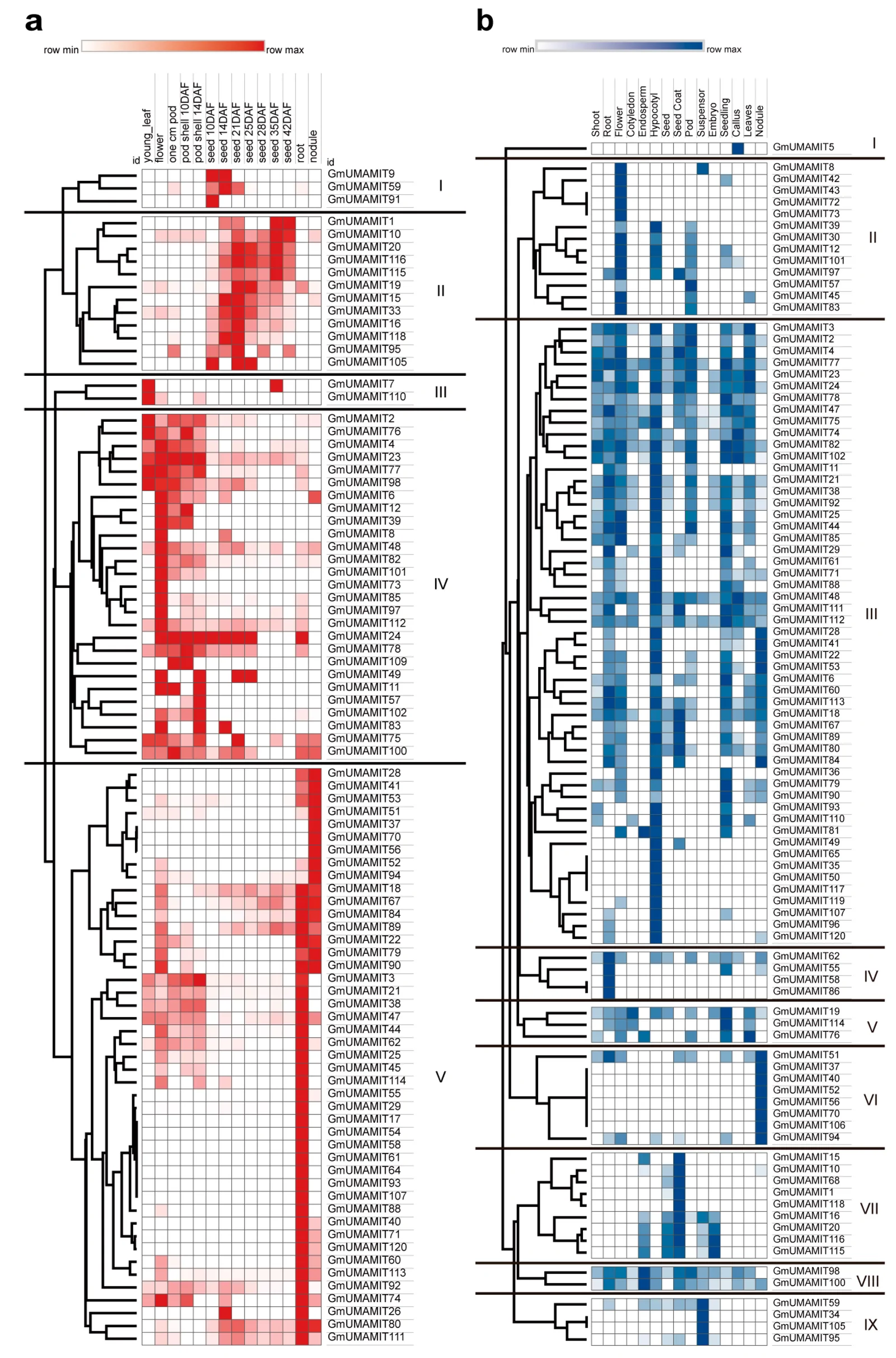
Expression patterns of GmUMAMIT genes in developmental stages and tissues of soybean. The color scale bars display the scaled expression levels (FPKM in (**a**), TPM in (**b**), scaled each gene by subtracting the median, divided by the standard deviation, the roman numerals represent different clusters). GmUMAMIT genes without expression in any of the cell types have been excluded from the figure. Detailed information is given in Table S8.

The tissue-specific expression profiles of GmUMAMIT genes were analyzed to provide insights into their physiological and biochemical functions. Based on available RNA-seq data from Soybase and the Soybean Expression Atlas, the temporal and spatial expression patterns of 120 GmUMAMIT genes were retrieved and are shown in Figure 4 and Table S8. The expression profiles of 14 tissues and developmental stages (young leaf, flower, one cm pod, pod shell at 10 DAF, pod shell at 14 DAF, seed at 10 DAF, seed at 14 DAF, seed at 21 DAF, seed at 25 DAF, seed at 28 DAF, seed at 35 DAF, seed at 42 DAF, root, and nodule) in Figure 4a were derived from Soybase. Eighty-nine GmUMAMIT genes were divided into clusters I–V using hierarchical clustering. The largest cluster of genes (cluster V in Figure 4a) was mainly expressed in roots or nodules. Several members showed tissue-specific expression; for example, *GmUMAMIT37*, *GmUMAMIT70*, and *GmUMAMIT56* were exclusively expressed in nodules, whereas *GmUMAMIT17*, *GmUMAMIT54*, *GmUMAMIT58*, *GmUMAMIT61*, *GmUMAMIT64*, *GmUMAMIT93*, and *GmUMAMIT107* were exclusively expressed in roots. The Soybean Expression Atlas integrated and normalized data from 1298 publicly available soybean transcriptome samples and quantified gene expression levels across 15 different tissue categories (shoot, root, flower, cotyledon, endosperm, hypocotyl, seed, seed coat, pod, suspensor, embryo, seedling, callus, leaves, and nodule). We retrieved tissue expression levels of GmUMAMITs from this dataset. Ninety-seven GmUMAMITs were divided into clusters I–IX (Figure 4b). Members of the largest cluster (cluster III in Figure 4b) showed the highest expression levels in hypocotyl, whereas other tissues exhibited varying expression levels.

The tissue-specific expression patterns were maintained consistently between the two datasets. Cluster II in Figure 4a roughly corresponded to cluster VII in Figure 4b, including *GmUMAMIT115*, *116*, and *118*, which were specifically expressed in seed tissues and may be involved in seed protein accumulation. Cluster IV in Figure 4a corresponded roughly to cluster III in Figure 4b (e.g., *GmUMAMIT4*, *23*, and *77*) or to cluster II (e.g., *GmUMAMIT12*, *39*, and *8*), whereas cluster V in Figure 4a roughly corresponded to cluster III (e.g., *GmUMAMIT28*, *41*, and *53*) or to cluster VI (e.g., *GmUMAMIT51*, *37*, and *70*) in Figure 4b. When analyzed in conjunction with phylogenetic trees, we found that most members of clades B and C were grouped into cluster V in Figure 4a and cluster III in Figure 4b, indicating higher expression levels in roots, nodules, or hypocotyls than other tissues, but were not expressed in the endosperm or suspensor. The difference was that most members of clade B were expressed in the embryo but not in the seed coat, whereas most members of clade C were not expressed in the embryo. Although the tissue expression patterns of clade D members were diverse, they exhibited great tissue specificity at the single-gene level.

### 2.6. Analysis of QTLs Around GmUMAMIT Genes

**Figure 5:**
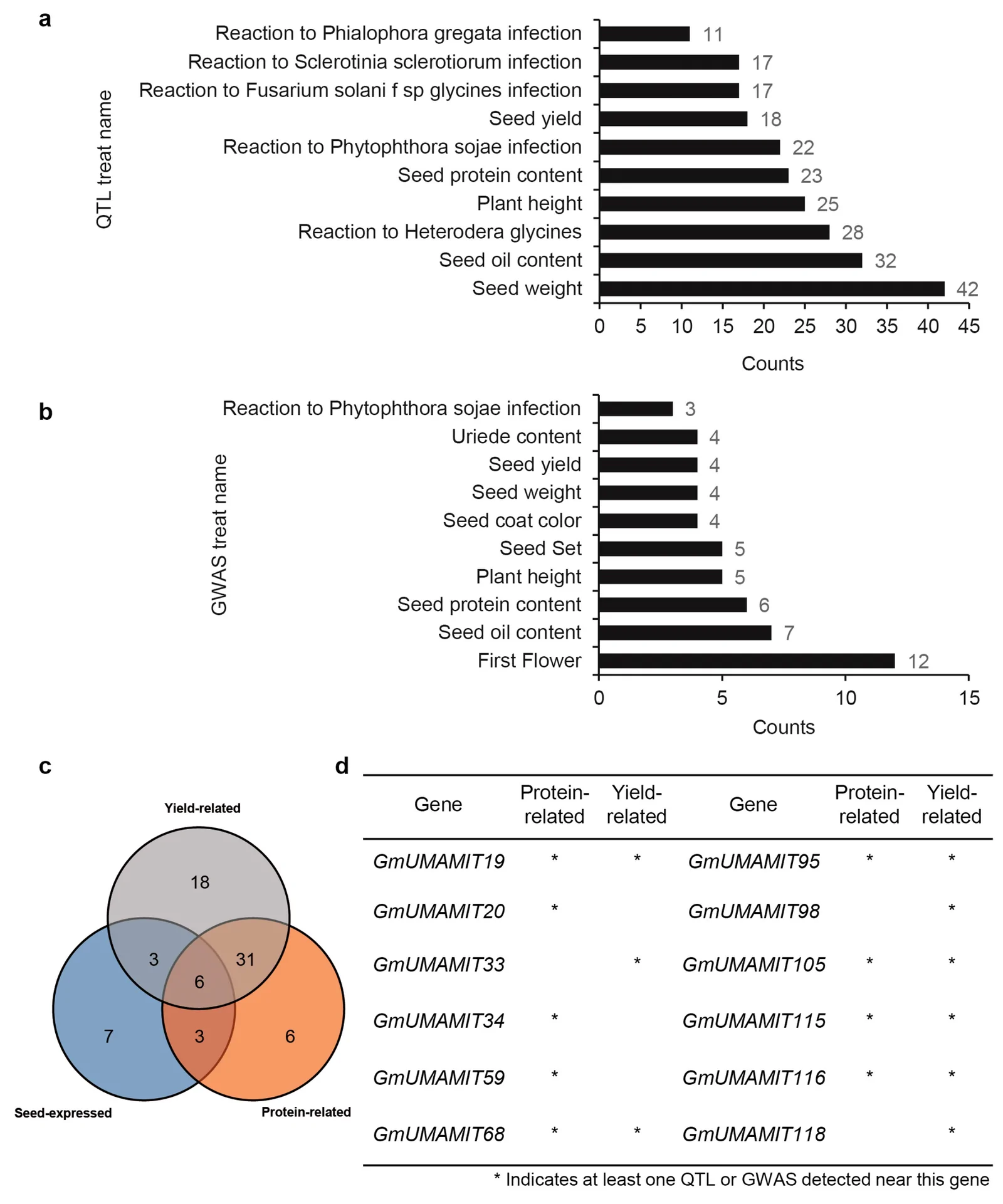
QTLs and GWAS loci around GmUMAMIT genes. (**a**) Analysis of QTLs detected near GmUMAMIT genes, with data from Soybase; (**b**) analysis of GWAS loci detected near GmUMAMIT genes, with data from Soybase; (**c**) a Venn diagram for GmUMAMIT genes specifically expressed during seed development (Figure 4a clusters I, II and Figure 4b classes VII, VIII, IX), GmUMAMIT genes containing yield-related QTLs or GWAS loci (seed weight, seed yield, seed size and seed number), and GmUMAMIT genes containing protein-related QTLs or GWAS loci (seed protein and seed amino acids); (**d**) candidate genes, where an asterisk indicates the presence of related QTLs or GWAS near the gene. Detailed information is given in Table S9.

To explore the possible roles of GmUMAMIT members in soybean agronomic traits, we searched for QTLs based on the chromosomal location of GmUMAMIT genes. Soybase contains more than 4800 bi-parental QTLs, of which 618 were associated with GmUMAMIT genes. In addition, 100 of more than 2800 genome-wide association study (GWAS) loci were located near GmUMAMIT genes (Table S9). We analyzed the trait categories corresponding to these QTLs and GWAS loci (Figure 5a,b). GmUMAMIT genes were mainly associated with seed weight, yield, seed protein, seed oil content, plant height, and pathogen resistance. 

We combined members that were primarily expressed during seed development (Figure 4a, clusters I and II, and Figure 4b, clusters VII, VIII, and IX) and members containing QTL loci related to seed weight, yield, and protein traits to create a Venn diagram to identify candidate genes (Figure 5c,d). A total of 12 candidate genes were identified. Among these genes, *GmUMAMIT19* exhibited higher expression levels during seed development stages and contained several QTLs related to protein and yield (*Seed protein 21-9*; *Seed protein 36-37*; *Seed yield 2-g4*; *Seed yield 5-g1*; *Seed weight 8-g3*). *GmUMAMIT20* was the most highly expressed gene in the late stage of seed development and contained a protein QTL (*Seed protein 27-4*). *GmUMAMIT59* was the most highly expressed gene in early seed stages, which may be related to seed lysine (Lys) content (*Seed Lys 3-1*). *GmUMAMIT95* was mainly expressed in the suspensor, and the surrounding genomic region was a hotspot of GWAS loci for seed protein and amino acid content. *GmUMAMIT118* was exclusively expressed in the seed coat and contained a yield-related QTL locus (*Seed yield 31-8*).

### 2.7. qPCR for Candidate GmUMAMIT Genes

**Figure 6:**
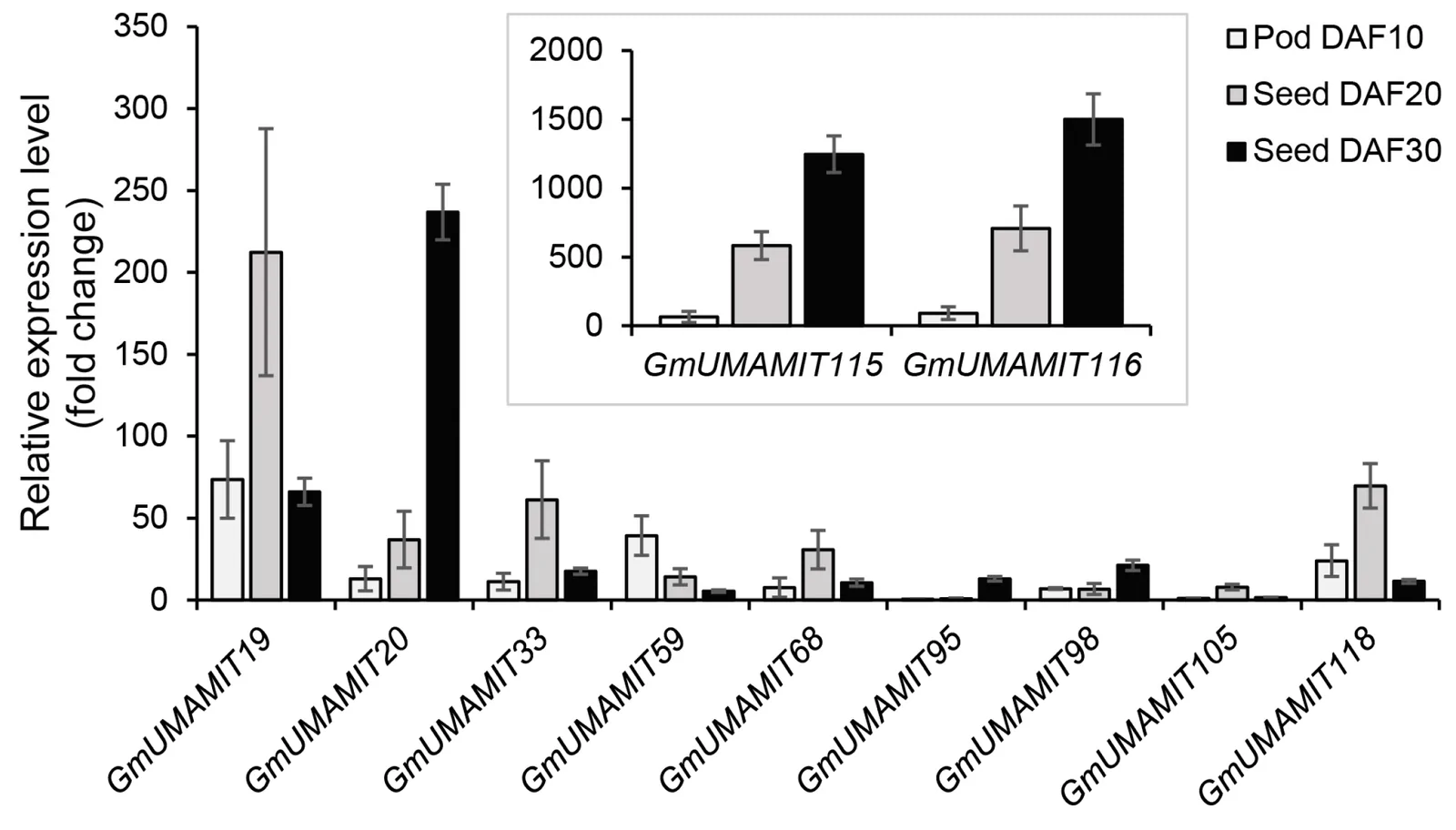
Relative gene expression of candidate *GmUMAMITs* in soybean seed development stages. The pods/seeds of soybean cultivar W82 in DAF10, DAF20 and DAF30 were used for analysis. Bar represents the mean ± standard deviation of three biological repeats and three technical repeats.

Pods from cultivar Williams 82 (W82) were harvested at 10, 20, and 30 days after flowering (DAF), and qPCR was used to quantify expression levels to confirm the expression of 12 candidate genes during developing seed stages (Figure 6). *GmUMAMIT34* was not detected due to its low expression level. Notably, compared to other genes, the expression levels of *GmUMAMIT115* and *GmUMAMIT116* in seeds at DAF 20 and DAF 30 were dramatically higher. These genes exhibited the highest expression levels in the endosperm, seed, and embryo in Figure 4b. *GmUMAMIT19* was the most highly expressed gene in pods at DAF 10 and showed high expression levels in seeds at DAF 20. In addition, *GmUMAMIT20* had high expression levels in seeds at DAF 30.

**Figure 7:**
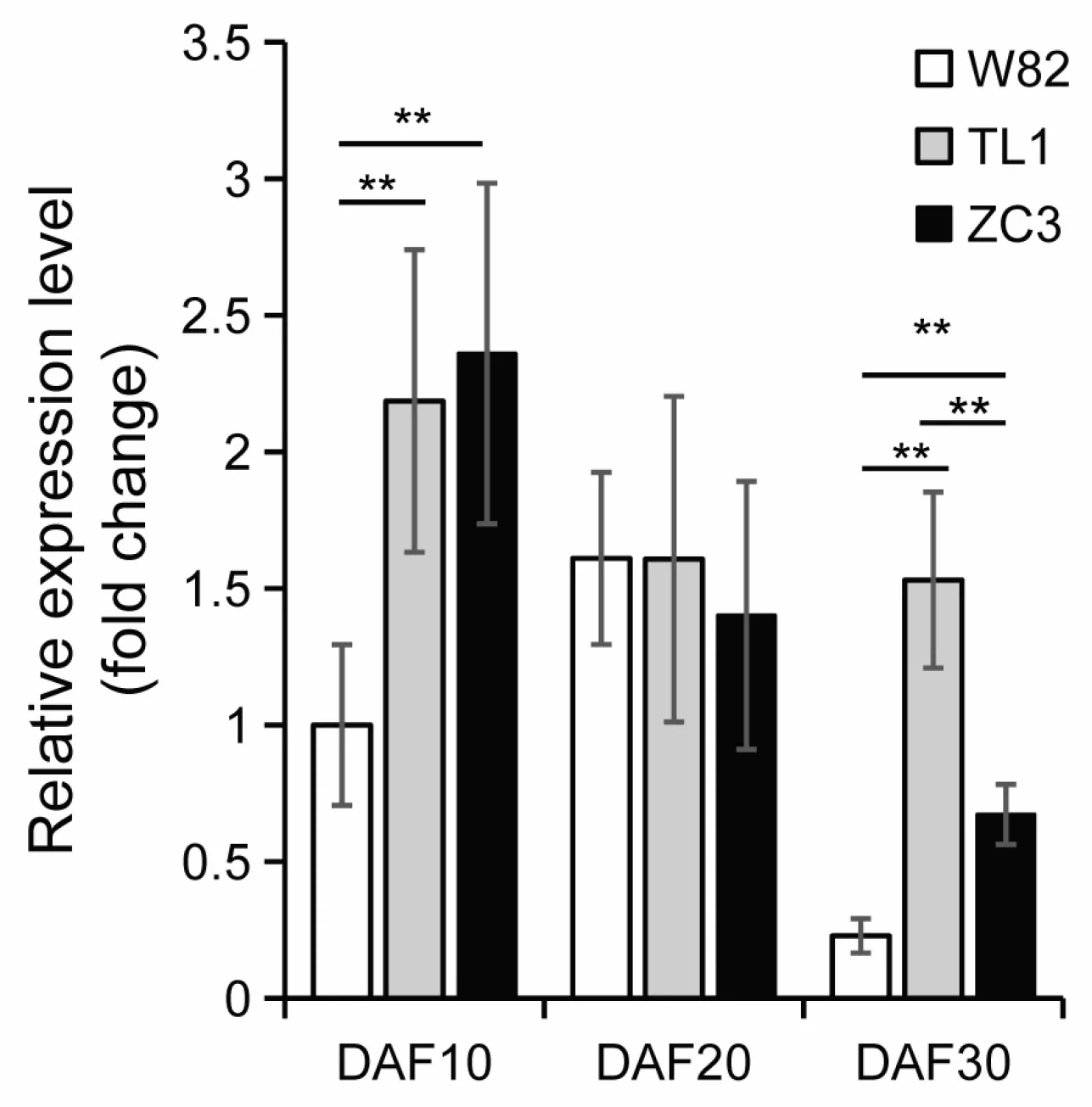
Relative gene expression of *GmUMAMIT118* in soybean seed development stages. The pods/seeds of soybean cultivars W82, TL1 and ZC3 in DAF10, DAF20 and DAF30 were used for analysis. Bar represents the mean ± standard deviation of three biological repeats and three technical repeats. Asterisks represent significant differences according to Turkey’s test (*p* < 0.01, **).

To prioritize candidates among the twelve potential genes, we compared their expression in developing seeds of W82, TL1, and ZC3 (seed protein content: 36.0%, 38.2%, and 42.7%, respectively). As shown in Figure 7, *GmUMAMIT118* exhibited a consistent positive correlation with seed protein content. The *GmUMAMIT118* expression levels of W82, TL1, and ZC3 in pods at DAF 10 were consistent with seed protein content levels. The expression levels of TL1 and ZC3 in seeds at DAF 30 were still significantly higher than those in W82, despite the fact that there was no significant difference among the three cultivars in seeds at DAF 20.

### 2.8. Functional Characterization of GmUMAMIT118 in Transgenic Arabidopsis

**Figure 8:**
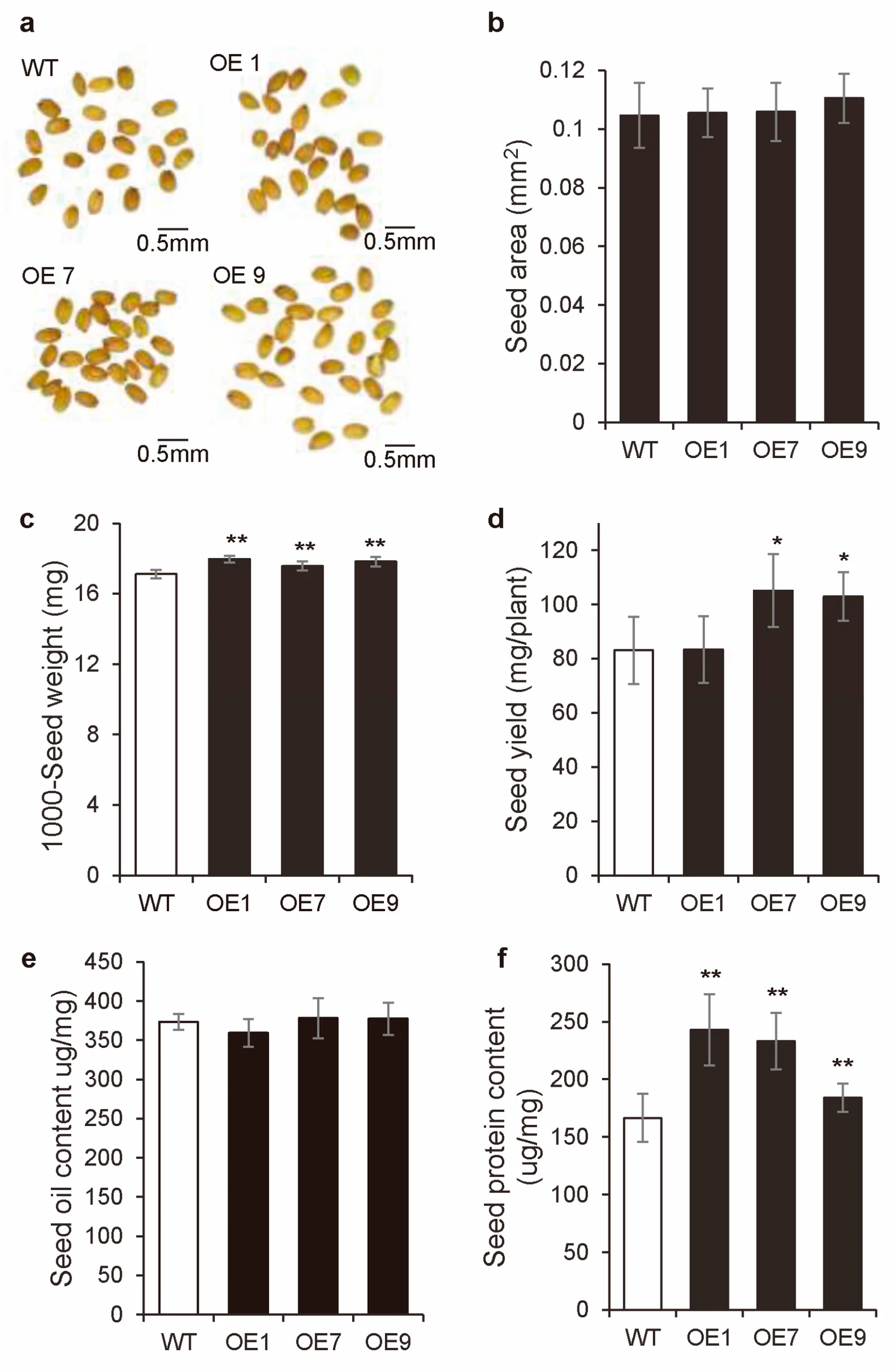
Phenotypes of *GmUMAMIT118*-overexpressing Arabidopsis plants. (**a**) seed phenotypes, (**b**) seed area (**c**), 1000-seed weight (**d**), seed yield per plant, (**e**) seed oil content, and (**f**) seed soluble protein content. Scale = 0.5 mm; *n* = 12. Error bars represent the mean standard deviation; significance analysis is based on Student’s *t*-test. 0.05 < *p* <0.01, *; *p* < 0.01, **.

We analyzed the seed phenotypes of *GmUMAMIT118*-overexpressing *Arabidopsis thaliana* lines and wild-type plants to better understand the role of *GmUMAMIT118* (Figure 8). Although the seeds of overexpression lines were only slightly larger than those of the wild type, the thousand-seed weight significantly increased. Analysis of yield per plant further demonstrated that overexpressing *GmUMAMIT118* in *A. thaliana* significantly improved yield. The protein content of seeds increased, whereas the oil content remained constant. These findings reveal that *GmUMAMIT118* is a promising target for increasing seed protein content while maintaining oil content and enhancing yield.

## 3. Discussion

In this study, we systematically identified and characterized members of the soybean UMAMIT family through phylogenetic reconstruction, evolutionary event analysis, tissue expression profiling, and QTL/GWAS data mining. These comprehensive data provide a framework for the GmUMAMIT family and serve as a reference for researchers interested in selecting specific UMAMIT members for functional characterization. In addition, the candidate genes identified here provide targets for improving soybean seed protein content and offer insights into molecular breeding of high-protein soybean varieties.

### 3.1. Characterization of the GmUMAMIT Family Provides Insights into Evolution and Function

A total of 120 UMAMIT genes were identified in soybean, more than in any other species in this study and nearly twice as many as in *M. truncatula* (78) and *P. vulgaris* (62), which are also legumes. The UMAMIT family size was not correlated with genome size but was significantly correlated with the number of protein-coding genes (Figure S2). Soybean is a paleotetraploid, with nearly 75% of its genes present in multiple copies [53]. WGD was the main driving force behind GmUMAMIT gene duplication, a pattern consistent with that observed for other gene families in soybean, such as the AAT gene family [55], the GSK3 gene family [56], and others. Collinearity analysis of three legume species revealed that GmUMAMIT genes not only duplicated during the recent Glycine WGD but also retained more genes during the legume WGD compared to *M. truncatula* and *P. vulgaris*. All proteins consisted only of EamA domains, and most contained 10 transmembrane domains. Earlier studies of Drug/Metabolite Exporter (DME) families showed that this structure was derived from the tandem duplication of five-TMD ancestor proteins [19]. Given the diversity of transport substrates, including amino acids, it is understandable that topological structure was more conserved than amino acid residues. 

The phylogenetic tree constructed in this study grouped UMAMIT proteins into clades A–J, consistent with previous findings by Zhao [43]. GmUMAMIT proteins were predominantly distributed outside clade H, whereas clades C, D, and J contained the largest number of UMAMIT members. This pattern was also observed in the other two legume species, *M. truncatula* and *P. vulgaris*. The difference with previous findings is that several sequences from early-diverging land plants—including *P. patens*, *S. moellendorffii*, *M. polymorpha*, *A. filiculoides*, and *S. cucullata*—were not placed in clade A but grouped together, reflecting high sequence divergence among these lineages. Clade H was not restricted to conifers; it also included two members from the basal angiosperm *Nymphaea colorata* (ANA-grade). 

### 3.2. Potential of Candidate GmUMAMITs to Improve Soybean Seed Traits

Achieving a higher yield without sacrificing seed oil and protein content has long been a goal of soybean breeding, but achieving this goal remains challenging. SWEETs and UMAMITs unload sucrose and amino acids in the phloem, respectively, whereas SUCs and AAPs are responsible for phloem loading [44,45,46]. *PsAAP1* overexpression in peas increased yield per plant and total seed protein through enhanced amino acid source-to-sink allocation. Notably, no differences were observed in starch content in *PsAAP1*-overexpressing peas [14]. Overexpression of *GmAAP6a* in soybeans improved tolerance to low nitrogen levels and increased seed protein content [17]. Therefore, manipulating amino acid transporters represents a promising strategy to overcome the yield–protein trade-off in soybean breeding. Here, we identified 120 UMAMIT genes in the soybean genome, and obtained 12 candidate genes by integrating expression profiles with QTL and GWAS datasets. However, the broad genomic intervals inherent to linkage and GWAS analyses often harbor multiple genes, limiting these approaches to candidate gene discovery rather than functional elucidation. Intriguingly, while our analyses linked GmUMAMIT members to pathogen resistance, yield, and seed quality—paralleling functional reports for AtRTP1 [35] and AtSIAR1 [28]—they did not capture the well-documented roles of amino acid transporters in NUE or low-nitrogen responses [14,15,17]. To address this discrepancy, we suggest leveraging complementary datasets, including publicly available transcriptomes from nitrogen starvation treatments, to probe the environmental responsiveness of this gene family.

Among the candidates, *GmUMAMIT118* emerged as particularly compelling. Its expression correlated positively with seed protein content across cultivars with divergent protein phenotypes (W82 < TL1 < ZC3), and its heterologous overexpression in Arabidopsis recapitulated the desired agronomic profile: elevated seed protein and yield without detectable alterations in oil content. Nevertheless, due to fundamental physiological differences between Arabidopsis and soybean, these results require direct experimental validation in soybean. Furthermore, these findings justify a germplasm-wide survey to assess whether natural allelic variants of *GmUMAMIT118* are associated with seed protein content.

Additionally, phylogenetic and collinearity analyses revealed that *GmUMAMIT20*, *GmUMAMIT68*, *GmUMAMIT115*, *GmUMAMIT116*, and *GmUMAMIT118* are all homologous to *AtUMAMIT25* (Figure 1 and Table S7). All five genes are candidate genes. Collinearity analysis showed that *GmUMAMIT20*, *GmUMAMIT68*, *GmUMAMIT115*, and *GmUMAMIT118* were retained as duplicated gene pairs in both the legume WGD and the Glycine WGD. These homologous genes shared a high level of protein sequence similarity (~86%), and *GmUMAMIT115* and *GmUMAMIT116* have identical protein sequences, with only one synonymous nucleotide substitution in the coding sequence. According to hierarchical clustering of tissue-specific expression data, all five genes were grouped into cluster II in Figure 4a or cluster VII in Figure 4b. Members of these two clusters are mainly expressed in seeds. These five genes had noticeably higher expression levels in developing seeds than other candidate genes, as determined by qPCR. In the vicinity of these five genes, several QTL/GWAS loci were discovered for traits related to yield and seed protein content. *GmUMAMIT20* was located near the QTL *Seed protein 27-4*; *GmUMAMIT68* was in the region of the QTL *Seed yield 31-8*, *Seed protein 36-39*, *Seed weight 37-6*, and *Seed weight 12-2*; *GmUMAMIT115/116* were located close to the QTL *Crude protein* and *R6 1-7*; and the QTL *Seed number 2-1* was located near the *GmUMAMIT118* locus. Despite subtle variations in tissue expression patterns, their high sequence conservation and duplication events probably suggest functional redundancy in seed amino acid transport. Consequently, single-gene manipulation may yield incomplete phenotypic resolution. To dissect the relative contributions of these genes and fully unlock their breeding potential, we propose leveraging CRISPR-mediated multiplex genome editing to simultaneously target these GmUMAMIT genes. This approach parallels recent work in Arabidopsis, where the simultaneous targeting of *AtUMAMIT29-31* delineated their collective function as seed glucosinolate exporters [38].

Regarding other candidate genes, phylogenetic analysis grouped *GmUMAMIT19* and *AtUMAMIT14* into one cluster. Both genes are broadly expressed, with higher expression levels in developing seeds. *GmUMAMIT59* and *GmUMAMIT95* are homologous to *AtUMAMIT24*, with higher expression levels in the suspensor. The expression of *AtUMAMIT14* and *AtUMAMIT24* in the chalazal seed coat indicates their involvement in phloem unloading during seed development [29,30]. Limited available experimental data make it difficult to formulate similar hypotheses for the soybean homologs.

### 3.3. Other Hypothetical Roles of GmUMAMITs

AtWAT1/AtUMAMIT5 was identified as an auxin transporter, and the mutant plant *atwat1* displayed secondary growth defects in stems [25,26]. SiWAT1, a tomato homolog, is involved in auxin homeostasis in the vascular cambium of woody tissues [27]. This is consistent with the fact that members of the AUX subfamily, which belongs to the AAT family, are auxin transporters [57]. According to the phylogenetic tree, AtUMAMIT5 was grouped into clade B, and GmUMAMIT21, 38, 92, and 107 are the soybean homologs of AtUMAMIT5, sharing identical domains, motifs, TMDs, and intron–exon organization. Despite being located on different chromosomes, these genes share a high degree of protein sequence similarity (88.72–97.44%). The ancestral gene was duplicated in both the legume WGD and the Glycine WGD, with two paralogous pairs identified: *GmUMAMIT21/38* and *GmUMAMIT92/107*. Analysis of tissue-specific expression showed that the four genes were grouped into cluster V in Figure 4a and cluster III in Figure 4b, among which *GmUMAMIT107* was specifically expressed in roots, hypocotyls, and seedlings, while *GmUMAMIT21*, *38*, and *92* were highly expressed in roots or hypocotyls and also widely expressed in other tissues. These findings support the hypothesis that GmUMAMIT21, 38, 92, and 107 function redundantly as auxin transporters in roots.

Pathogen resistance is another function of UMAMITs, such as AtWAT1, AtRTP1, and AtUMAMIT20 [34,35,37]. Similar functions have been widely reported in the AAT gene family [58,59]. QTL and GWAS data in the vicinity of GmUMAMITs also point to a widespread role for GmUMAMITs in pathogen resistance. AtRTP1/AtUMAMIT36 and soybean homologs GmUMAMIT45 and GmUMAMIT46 were grouped into clade J. *GmUMAMIT45* and *GmUMAMIT46* are a recent tandem duplication product. Both genes are identical in TMD number and motif composition and exhibit high sequence similarity. Each gene contains two EamA domains, although one EamA domain in *GmUMAMIT46* is non-significant. *GmUMAMIT45* was expressed in flowers and pods, whereas no expression data were available for *GmUMAMIT46*. These findings support the hypothesis that GmUMAMIT45, like RTP1, is involved in pathogen resistance, whereas GmUMAMIT46 may have rapidly lost function.

## 4. Materials and Methods

### 4.1. Identification and Characterization of UMAMIT Genes in Soybean

The soybean genome (Wm82.a2.v1) sequence and annotation GFF3 file were downloaded from Phytozome 12 (https://phytozome.jgi.doe.gov, accessed on 2 December 2022) [53]. BLASTP searches were performed using 46 *Arabidopsis thaliana* UMAMIT protein sequences [43] as queries. Meanwhile, the Hidden Markov Model (HMM) profile of the EamA domain (PF00892) was downloaded from Pfam (http://pfam.xfam.org/, accessed on 2 December 2022), and an HMM search was performed using HMMER in the soybean protein database. All protein sequences were submitted to Pfam and NCBI CD-search to remove sequences lacking a significant EamA domain (E-value ≤ 1 × 10^−5^) or with incomplete domain coverage (<70%). *GmUMAMIT* genes were named according to their chromosomal locations. Protein lengths, molecular weight, and isoelectric point were calculated using ExPASy (https://web.expasy.org/protparam/, accessed on 10 December 2022). Subcellular localization of GmUMAMIT proteins was predicted via Plant-mPLoc (http://www.csbio.sjtu.edu.cn/bioinf/plant-multi/, accessed on 10 December 2022) [60].

### 4.2. Multiple Sequence Alignment and Phylogenetic Analysis

To investigate the phylogenetic relationships of the UMAMIT family, we searched for UMAMIT members in the genome of *Marchantia polymorpha* v3.1 [61], *Azolla filiculoides* v1.2, *Salvinia cucullata* v1.2 [62], *Nymphaea colorata* v1.2 [63], *Zea mays* RefGen V4 [64], *Medicago truncatula* Mt4.0v1 [65], *Phaseolus vulgaris* v2.1 [66], and *Oryza sativa* 7.0 [67] using the same method described above. Together with UMAMIT proteins of *Physcomitrella patens*, *Selaginella moellendorffii*, *Picea abies*, *Pinus pinaster* and *Arabidopsis thaliana*, we constructed a phylogenetic tree [43]. All sequences were aligned using MAFFT [68], and trimAL [69] was used to trim the alignment sequences. A maximum likelihood (ML) phylogenetic tree was constructed using IQ-Tree [70]. The best-fit model was selected (JTT+F+R7), and the UltraFast Bootstrap options were selected for bootstrap analysis with 5000 replicates.

### 4.3. Analysis of Conserved Domain, Motif, and Gene Structure

Conserved domain information of GmUMAMIT members was retrieved from Pfam. CCTOP was used to predict the transmembrane domains (http://cctop.ttk.hu/, accessed on 22 December 2022) [71]. Conserved motifs were analyzed using MEME (https://meme-suite.org/meme/tools/meme, accessed on 22 December 2022) [72] with a maximum number of motifs of 10 and a motif length of 15–30 amino acids. Information on gene structure was extracted from the GFF3 file to show the exons, introns, and UTRs of UMAMIT genes.

### 4.4. Synteny Analysis and Ka/Ks Ratio

Gene duplication events were analyzed using the Multiple Collinearity Scan toolkit (MCScanX) implemented in TBtools-IIv.2.485 [73,74]. The ratio of nonsynonymous substitutions per nonsynonymous site (Ka) to synonymous substitutions per synonymous site (Ks) was estimated using the “Simple Ka/Ks Calculator” function in TBtools [74]. Subsequently, the Ks values were used to calculate the estimated date of each duplication event (T = Ks/2λ), assuming a synonymous substitution rate (λ) of 6.1 × 10^−9^ [75].

### 4.5. Analysis of Expression Patterns and QTLs Around GmUMAMIT Genes

To analyze the tissue expression patterns of GmUMAMITs, RNA-seq data were downloaded from Soybase (https://www.soybase.org/, accessed on 9 January 2023) and the Soybean Expression Atlas (https://venanciogroup.uenf.br/cgi-bin/gmax_atlas/, accessed on 11 January 2023) [76], and then visualized as a heatmap.

The reported QTL/GWAS loci in soybean were downloaded from Soybase. Based on the Wm82.a2.v1 genome annotation, QTLs flanking GmUMAMIT genes (within 10 kb upstream and downstream of GWAS QTL) were anchored to the corresponding physical positions of the genome.

### 4.6. Plant Materials and Growth Conditions

*Arabidopsis thaliana* ecotype Columbia (Col-0) and *Glycine max* cultivars Williams 82 (W82), Zhechun 3 (ZC3), and Tianlong 1 (TL1) were routinely stored in 4 °C and used for all experiments. Col-0 and W82 seeds were obtained from the Institute of Genetics and Developmental Biology, Chinese Academy of Sciences (Beijing, China). TL1 seeds were provided by the Oil Crops Research Institute, Chinese Academy of Agricultural Sciences (Wuhan, China), and ZC3 seeds were obtained from the Institute of Specialty Crops, Chongqing Academy of Agricultural Sciences (Chongqing, China).

Soybean seeds were germinated in a peat–vermiculite mixture (1:1, *v*/*v*) and grown under a 14 h light/10 h dark photoperiod at 22 °C, 60% relative humidity, and a light intensity of 150 µmol·m^−2^s^−1^. Seedlings were transplanted to the field in Chongqing, China (106.42° E, 29.82° N) two weeks post germination in April and maintained with regular irrigation. Tissues (pods at 10, 20, and 30 days after flowering [DAF]) were collected, frozen in liquid nitrogen, and stored at −80 °C for subsequent RNA isolation.

*Arabidopsis thaliana* ecotype Col-0 was used for *Agrobacterium tumefaciens*-mediated transformation experiments. Seeds were surface-sterilized with 10% sodium hypochlorite and then rinsed with sterile distilled water. After germinating on 1/2 MS medium, seedlings were transplanted into pots containing a mixture of peat, vermiculite, and perlite (6:3:1), and grown under a 16 h light/8 h dark photoperiod, 25/18 °C day/night temperature, 60% humidity, and a light intensity of 120 µmol m^−2^s^−1^.

### 4.7. RNA Isolation and Real-Time Quantitative PCR

Total RNA was isolated using the Biospin Plant Total RNA Extraction Kit (Bioer Technology, Hangzhou, China), and first-strand cDNA was synthesized using PrimeScript™ II 1st Strand cDNA Synthesis Kit (TaKaRa Biotechnology, Dalian, China) according to the manufacturer’s protocol. All primers used for qRT-PCR were tested for specificity and amplification efficiency (Table S10). Reactions were performed using Hieff qPCR SYBR Green Master Mix (Yeasen Biotechnology, Shanghai, China) according to the manufacturer’s guidelines and run on a QuantStudio 3 (Thermo Fisher Scientific, Waltham, MA, USA), with three biological and technical replicates. Relative expression levels were calculated using the 2^−∆∆Ct^ method.

### 4.8. Vector Construction and Plant Transformation

Total RNA was isolated from developing seeds of soybean cultivar ZC3, and cDNA was synthesized using PrimeScript™ II 1st Strand cDNA Synthesis Kit (TaKaRa Biotechnology, Dalian, China). The coding sequence of *GmUMAMIT118* (*Glyma.20G094100*) was amplified and cloned via In-Fusion into the plant binary vector pCAMBIA1300-GFP, resulting in the p35S::*GmUMAMIT118*-GFP vector. The vector was introduced into *Agrobacterium tumefaciens* strain GV3101, and *Arabidopsis thaliana* (Col-0) plants were transformed using the floral dip method. Transgenic plants were selected on 1/2 MS medium containing 25 mg/L hygromycin and further confirmed by PCR.

### 4.9. Quantification of Seed Traits

Mature Arabidopsis seeds were collected for yield analysis. Total oil was isolated and quantified using the hexane extraction method [77], with three biological replicates. Briefly, dried seeds were ground into powder. The powder was soaked in hexane overnight; the supernatant was collected by centrifugation. The amount of the supernatant after complete volatilization was calculated for seed oil content.

Total soluble protein content was determined using a plant protein extraction kit (Solarbio Life Sciences, Beijing, China) and a BCA protein concentration kit (Solarbio Life Sciences, Beijing, China). In brief, dried seeds were finely ground into powder with liquid nitrogen. The powder was mixed with 1 mL of lysis buffer supplemented with 10 µL protease inhibitor cocktail and 10 µL PMSF and incubated at 4 °C for 20 min, with shaking every 5 min. After centrifugation, the supernatant was used for total protein concentration determination by using the bicinchoninic acid (BCA) assay, using a Multiskan GO microplate reader (Thermo Fisher Scientific, Waltham, MA, USA).

## 5. Conclusions

In conclusion, we identified and characterized 120 UMAMIT family members in soybean, as well as their phylogenetic relationships, evolutionary events, tissue expression patterns, and QTL/GWAS data, which provide a comprehensive resource for functional studies of amino acid transporters. In addition, twelve UMAMITs were identified as candidates for improving seed protein content and yield. Compared with the wild type, *GmUMAMIT118*-overexpressing *Arabidopsis thaliana* showed increased yield and seed protein content. Further studies are warranted to functionally characterize *GmUMAMIT118* and other GmUMAMIT candidate genes in soybean, thereby providing valuable insights for molecular soybean breeding.

## Data Availability

The original data presented in this study are included in the article and Supplementary Materials. Further inquiries can be directed to the corresponding author.

## Supplementary Materials

The following supporting information can be downloaded at https://www.mdpi.com/article/10.3390/ijms27167079/s1.
