# Supplementary material for: Genome-Wide Identification of the Soybean UMAMIT Family and Functional Analysis of GmUMAMIT118 in Improving Seed Protein Content
Source: Int J Mol Sci. 2026 Aug 7;27(16):7079. doi: 10.3390/ijms27167079 (PMC13512864; doi:10.3390/ijms27167079)
Supplement: Supplementary file 1 [file ijms-27-07079-s001.zip › Supplementary Figures.pdf]

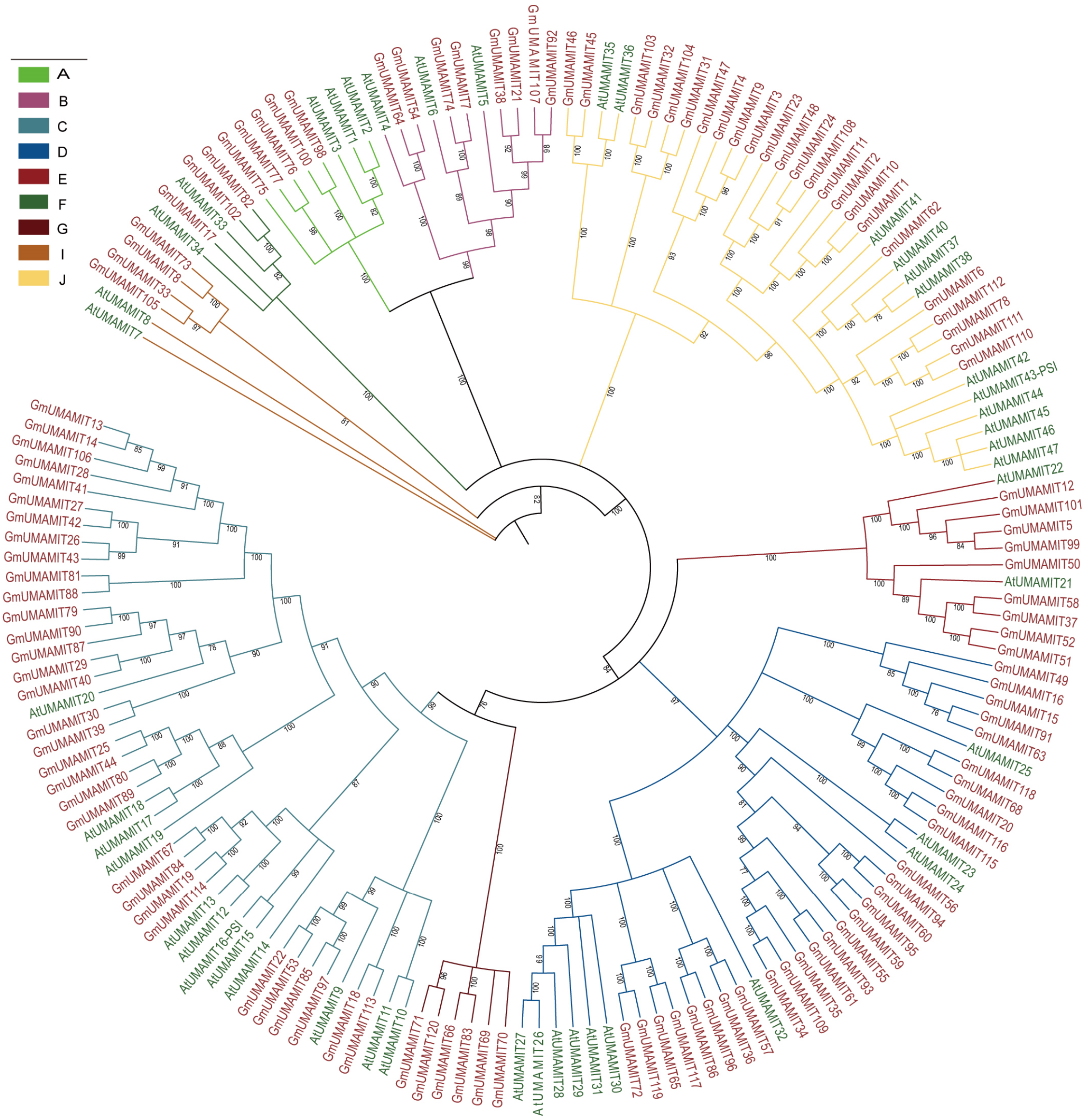

Figure S1. Phylogenetic tree analysis of the UMAMIT proteins in *Glycine max* and *Arabidopsis thaliana*. The gene IDs are abbreviated as; Gm, *Glycine max*; At, *Arabidopsis thaliana.*

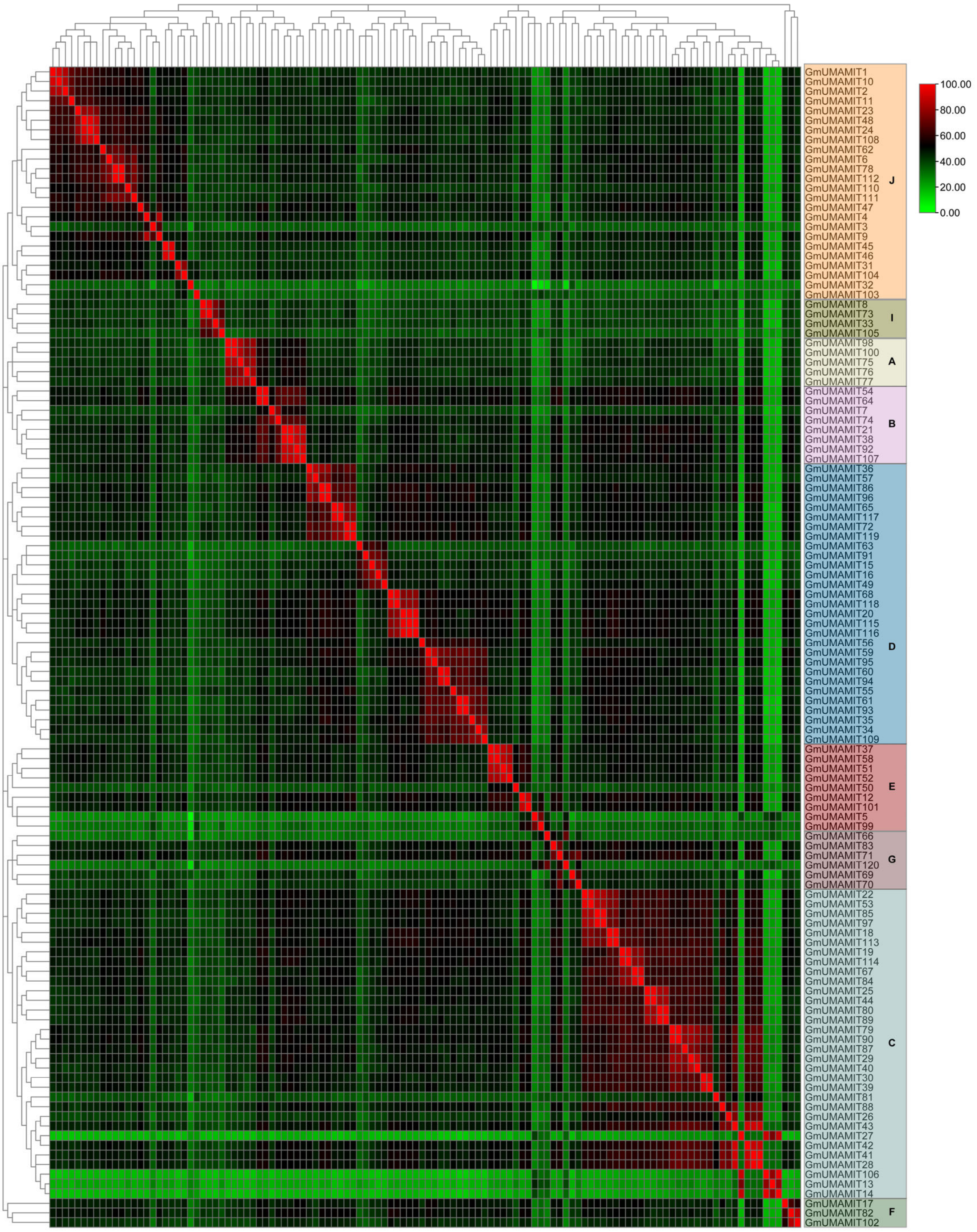

Figure S2. Heat map of protein sequence similarity among GmUMAMITs.

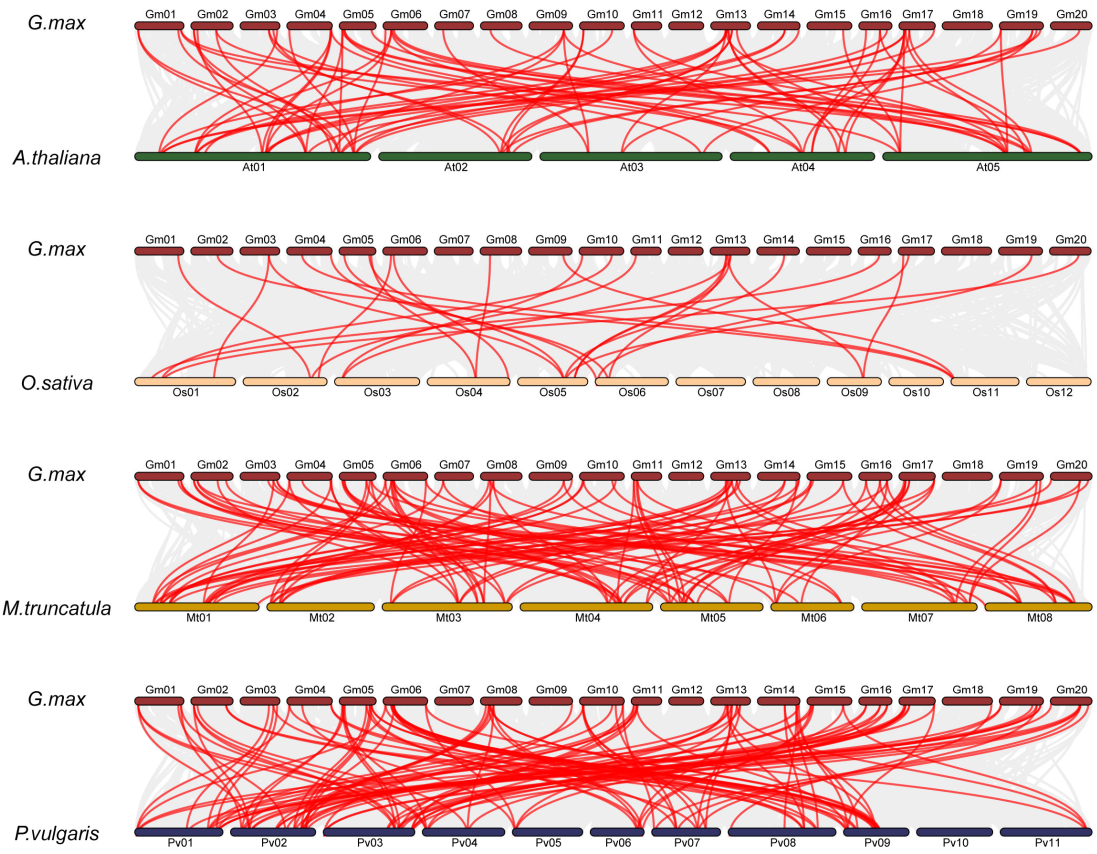

Figure S3. Inter-species synteny analysis between *Glycine max* and *Arabidopsis thaliana*, *Oryza sativa*, *Medicago truncatula*, and *Phaseolus vulgaris*. Grey lines indicated collinear relationships between *Glycine max* and other four species, the red lines highlighted syntenic UMAMIT gene pairs.
